# Low-Dose Paclitaxel Combined with Dehydrocavidine Synergistically Suppresses Cervical Cancer by Inhibiting FASN/SCD1-Mediated Lipid Metabolism

**DOI:** 10.3390/biom16091354

**Published:** 2026-09-17

**Authors:** Xuelian Luo, Jin Li, Tianjiao Xing, Shuang Liu, Yiting Deng, Hongxia Xu, Yu Xiao, Hongchen Wang, Xianing Peng, Ying Chen

**Affiliations:** College of Life Science, Yangtze University, No. 266 Jingmi Road, Jingzhou 434025, China

**Keywords:** cervical cancer, paclitaxel, dehydrocavidine, lipid metabolism, FASN, SCD1, drug synergy

## Abstract

Cervical cancer is a leading cause of cancer-related death among women, and acquired resistance to paclitaxel (PTX) frequently results in treatment failure. Dehydrocavidine (DHC), the principal bioactive alkaloid from the traditional Chinese herb *Corydalis saxicola*, has shown antitumor activity, but its potential in cervical cancer and synergism with PTX remain unknown. We investigated the combined effect of low-dose PTX and DHC in HeLa and SiHa cells and a HeLa xenograft model. ZIP (zero interaction potency) synergy analysis revealed strong synergy in inhibiting proliferation, colony formation, and migration. In vivo, the combination significantly suppressed xenograft tumor growth. Network pharmacology, molecular docking, and experimental validation identified fatty acid synthase (FASN) and stearoyl-CoA desaturase 1 (SCD1) as key targets; the combination downregulated their expression, reducing lipid droplets and triglyceride accumulation. Public single-cell transcriptomic analysis showed elevated FASN/SCD1 in tumor cells and cancer-associated fibroblasts, correlating with poor overall survival. These findings demonstrate that low-dose PTX plus DHC synergistically suppresses cervical cancer by inhibiting FASN/SCD1-mediated lipid metabolism reprogramming, providing a promising strategy to overcome PTX resistance and reduce toxicity.

## 1. Introduction

Cervical cancer ranks among the most frequently diagnosed gynecologic malignancies worldwide, and its pathogenesis is intimately linked to persistent infection with high-risk human papillomavirus (HPV) [1]. The Global Cancer Statistics 2024 report estimated that cervical cancer caused 604,000 incident cases and 280,000 deaths globally in 2024, ranking fifth in female cancer incidence and fourth in cancer-related mortality [2]. Patients with advanced cervical cancer commonly confront two major therapeutic challenges: acquired resistance to standard chemotherapeutic agents, which leads to diminished treatment efficacy [3], and severe chemotherapy-associated adverse effects—such as myelosuppression and gastrointestinal reactions—that frequently compromise patient compliance [4]. Over the past decade, the role of traditional Chinese medicine (TCM) as an adjunctive therapy in oncology has attracted growing interest, owing to its capacity to alleviate chemotherapy-induced toxicities, ameliorate hypercoagulable states, mitigate cardiotoxicity, and, to a certain degree, reverse resistance to radiotherapy and chemotherapy [5,6,7,8].

Paclitaxel (PTX) is a microtubule-stabilizing agent that promotes tubulin polymerization and blocks cell cycle progression at the G2/M transition, ultimately triggering apoptotic cell death [9]. Because of its broad-spectrum and potent antitumor activity, PTX is a standard therapeutic agent for a broad spectrum of cancers, such as breast, ovarian, non-small-cell lung, and Kaposi’s sarcoma [10]. Nevertheless, the clinical utility of PTX is also constrained by acquired drug resistance and neurotoxicity [11,12]. Consequently, the development of combination strategies that can sensitize tumors to PTX while reducing its toxicity has become an active area of investigation [13].

*Corydalis saxicola* Bunting, a plant belonging to the Papaveraceae family, is a traditional herbal medicine commonly used in the folk medicine of Southwest China [14]. It possesses the effects of clearing heat, eliminating dampness, dispersing blood stasis, and reducing swelling, and has been traditionally employed for the treatment of hepatitis, liver cirrhosis, and sores [14,15]. Modern pharmacological studies have demonstrated that extracts of *C. saxicola* and its active constituents exhibit multiple biological activities, including anti-inflammatory, antioxidant, and antitumor effects [14,15]. Dehydrocavidine (DHC), the principal isoquinoline alkaloid of *C. saxicola*, represents its main pharmacologically active substance [16]. Preclinical investigations have shown that DHC possesses a range of pharmacological activities, including immune response enhancement, tumor growth inhibition, and attenuation of chemotherapy-related toxicity, and has exhibited antitumor potential in models of tongue cancer and lung cancer [15,16,17]. Although DHC is extensively studied in Chinese medicinal research, its role in cervical cancer and the feasibility of its combination with PTX have, to date, not been systematically explored.

Against this background, the present study was designed to evaluate the combined effects of low-dose PTX in combination with DHC on cervical cancer cells in vitro and in vivo and to explore the underlying molecular mechanisms. Our results may provide a foundation for developing novel PTX-based combination regimens with improved efficacy and reduced toxicity.

## 2. Materials and Methods

### 2.1. Cell Culture and Reagents

Human cervical adenocarcinoma HeLa cells (Shanghai Institute of Biochemistry and Cell Biology, CAS, Shanghai, China) and cervical squamous carcinoma SiHa cells (Wuhan Servicebio Technology Co., Ltd., Wuhan, China) were maintained in DMEM containing 10% fetal bovine serum (Cat. No. G8003-100ML, Servicebio) and 1% penicillin-streptomycin (Cat. No. G4016-100ML, Servicebio) at 37 °C in a humidified 5% CO_2_ atmosphere. Paclitaxel (PTX) and dehydrocavidine (DHC) were prepared in DMSO and subsequently diluted to the desired working concentrations in culture medium.

### 2.2. Cell Viability, Dose–Response Curves, and Drug Synergy Analysis

Cell viability was assessed with the CCK-8 assay. HeLa and SiHa cells were seeded into 96-well plates (3 × 10^3^ cells/well), cultured overnight for attachment, and then exposed to single agents or combined PTX and DHC at the indicated concentrations for 48 or 72 h. After the treatment period, cells were incubated with serum-free medium containing 10% CCK-8 (Cat. No. SC119, Sevenbio) for 1 h at 37 °C in the dark. The optical density at 450 nm was measured, and viability was calculated as a percentage of that of vehicle-treated controls. GraphPad Prism 9.0 was used to generate dose–response curves and calculate the half-maximal inhibitory concentrations (IC_50_).

For synergy quantification, the ZIP (zero interaction potency) model implemented in SynergyFinder 3.0 (https://synergyfinder.fimm.fi, accessed on 10 March 2026) was employed. Viability measurements from single-drug and combination exposures were transformed into inhibition percentage matrices. The resulting ZIP synergy scores (δ values) were then derived, where δ > 0 indicates synergistic interactions, and δ > 10 designates strong synergy. The output heatmap uses red color saturation to represent the magnitude of synergy, and the algorithm automatically delineates the region with the highest δ scores. This high-synergy region guided the selection of the low-dose drug combination for all subsequent functional experiments.

### 2.3. Colony Formation and Wound Healing Assays

Clonogenic potential was evaluated by seeding HeLa or SiHa cells at low density (1 × 10^3^ cells/well) into 6-well plates. Following overnight attachment, cells were exposed to PTX (0.0025 μM), DHC (60 μM), or their combination for 48 h. The drug-containing medium was then replaced with complete medium without drugs, and cultures were maintained for up to 14 days, with the medium refreshed at 3-day intervals, until macroscopic colonies became apparent. After fixation with 4% paraformaldehyde, colonies were stained with 0.1% crystal violet. Clusters containing 50 or more cells were scored as a colony using ImageJ software (version 1.54f).

A scratch wound healing assay was performed to assess cell migration. Confluent cell monolayers were mechanically disrupted with a sterile 200 μL pipette tip to generate a uniform cell-free gap. Detached cells were removed by gentle PBS rinsing, and the remaining cells were incubated in serum-free DMEM containing the indicated drug formulations. Wound closure was monitored by capturing images of the same field at 0 h and 24 h post-scratch. The migration rate was quantified by measuring the reduction in wound area at 24 h relative to that at 0 h.

### 2.4. RNA Isolation, Quantitative Real-Time PCR, and Immunoblotting

Following 48 h of drug exposure, total RNA was isolated using TRIzol reagent (Cat. No. R411-01, Vazyme, Nanjing, China) following the supplier’s guidelines. Total RNA (1 μg) was converted to cDNA using the PerfectStart UniRT Kit (Cat. No. AQ211-01, TransGen, Beijing, China). Real-time qPCR was performed on a Bio-Rad CFX-96 instrument using TransStart Top Green qPCR SuperMix (Cat. No. AU341, TransGen). The relative mRNA expression of target genes was determined by the comparative threshold cycle (2^−ΔΔCt^) method, normalized to GAPDH. Oligonucleotide primers used in this study are provided in Table S1.

Whole-cell lysates were prepared by incubating harvested cells in cold RIPA buffer with protease inhibitors for 30 min on ice. The lysates were spun at 14,000× *g* (4 °C, 20 min), and the resulting cleared supernatants were analyzed for protein content using the BCA method (Cat. No. P0010, Beyotime, Shanghai, China). Following separation by 8% SDS-PAGE and wet transfer to PVDF (Millipore, Billerica, MA, USA), membranes were saturated with 5% non-fat dry milk in TBST (1 h, room temperature) and subsequently incubated with primary antibodies against FASN (1:1000; Cat. No. R013857, Epizyme, Shanghai, China), SCD1 (1:1000; Cat. No. R010079, Epizyme), and β-actin (1:50,000; Cat. No. 10494-1-AP, Proteintech, Rosemont, IL, USA) at 4 °C overnight. Following extensive washing, the membranes were reacted with horseradish peroxidase-linked secondary antibodies for 1 h at room temperature. Immunoreactive bands were detected using ECL substrate (Cat. No. P0018S, Beyotime) and recorded with a gel imaging system.

### 2.5. Oil Red O Staining and Triglyceride Assay

Oil Red O staining was employed to evaluate intracellular neutral lipid accumulation. After 48 h of designated treatments, a 15-min fixation step using 4% paraformaldehyde was applied, briefly rinsed with 60% isopropanol, and then incubated with freshly prepared Oil Red O working solution (Cat. No. G1015, Servicebio) for 20 min at ambient temperature. Following differentiation in 60% isopropanol and washes with distilled water, lipid droplets were visualized under a light microscope. The stained area was quantified using ImageJ software and data were normalized to the control and presented as percentages.

For triglyceride (TG) quantification, cells were pelleted, washed with PBS, and homogenized in RIPA lysis buffer. The resulting homogenate was directly employed for triglyceride measurement using a commercial enzymatic assay kit (Cat. No. A110-1-1, Nanjing Jiancheng Bioengineering Institute, Nanjing, China), with absorbance read at 500 nm. TG levels were normalized to total protein and expressed as μmol/g protein.

### 2.6. In Vivo Xenograft Model

Female BALB/c nude mice (4 weeks old) were obtained from GemPharmatech Co., Ltd. (Nanjing, China) and maintained in an SPF barrier facility under controlled conditions (12-h light/dark cycle, 22 ± 1 °C, 60–65% humidity) with ad libitum access to standard chow and water. After 3–5 days of acclimatization, HeLa cells (1 × 10^7^ in 200 μL PBS) were subcutaneously inoculated into the right axilla. Once tumors attained a volume of roughly 100 mm^3^, the animals were randomized into four groups (*n* = 3 per group) and received intraperitoneal injections of saline (control), PTX (5 mg/kg), DHC (50 mg/kg), or the combination of PTX (5 mg/kg) plus DHC (50 mg/kg), administered every other day. Tumor length (L) and width (W) were measured before each injection, and tumor volume was calculated as V = L × W^2^/2; body weight was recorded simultaneously. After 13 days of consecutive treatment, CO_2_ inhalation was used to euthanize the mice, and tumors were collected for weight measurement and photographic documentation. Each tumor was divided: one fragment was fixed in 4% formaldehyde and processed for paraffin embedding, while the remainder was flash-frozen in liquid nitrogen and kept at −80 °C. All animal procedures were approved by the Animal Ethics Committee of Yangtze University (Approval No. 202501097).

### 2.7. Network Pharmacology, Bioinformatics Analyses, and Molecular Docking

Potential therapeutic targets of paclitaxel (PTX) and dehydrocavidine (DHC) were systematically compiled from 11 publicly accessible databases (TCMSP, SwissTargetPrediction, PharmMapper, DrugBank, SymMap, HERB, SEA, PubChem, TTD, SuperPred, and DGIdb). After removal of redundant entries, only targets documented in at least two independent databases were retained, yielding 239 high-confidence targets for PTX and 69 for DHC. Cervical cancer-associated genes were sourced from six repositories (GeneCards, DisGeNET, OMIM, CTD, MalaCards, and TCGA) and subjected to the same filtration, resulting in a total of 11,968 disease-related genes. The intersection of these three datasets, depicted by a Venn diagram, identified 31 overlapping genes as candidate targets. Using STRING (v12.0, confidence ≥ 0.4), the protein–protein interaction (PPI) network was generated and then displayed in Cytoscape (v3.9.1). Functional characterization of the 31 shared targets was performed through Gene Ontology (GO) annotation and KEGG pathway enrichment analysis using the DAVID bioinformatics resource (v6.8), applying a significance cutoff of *p* < 0.05.

Comparative expression analysis of the candidate genes in cervical cancer and paired non-tumor tissues was examined via the GEPIA web server (http://gepia.cancer-pku.cn/, accessed on 18 March 2026), based on integrated TCGA and GTEx datasets, with screening criteria of |log_2_ fold change| > 1 and *p* < 0.01. The prognostic value of each gene was assessed by generating survival curves with the Kaplan–Meier Plotter platform (https://kmplot.com/analysis, accessed on 20 March 2026); patient cohorts were dichotomized by median expression values, and significance was determined by the log-rank test.

Molecular docking was performed to assess the binding potential of PTX and DHC toward FASN and SCD1. Three-dimensional protein structures were retrieved from the Protein Data Bank (PDB), and docking calculations were performed on the CB-Dock online platform (https://cadd.labshare.cn/cb-dock/, accessed on 23 March 2026). The resulting binding affinities, expressed in kcal/mol, were recorded for both ligand–protein pairs.

### 2.8. Single-Cell Transcriptomic and Functional Enrichment Analysis

Single-cell RNA sequencing (scRNA-seq) data were retrieved from the Cell-omics Data Coordinate Platform (CDCP, https://db.cngb.org/cdcp/, accessed on 25 March 2026). Publicly available cervical cancer datasets were queried, and the Gene Search module was employed to examine the expression profiles of FASN and SCD1 across distinct cell populations. The t-distributed stochastic neighbor embedding (t-SNE) method was employed for dimensionality reduction and subsequent cell-type annotation, allowing identification of specific cell subsets with elevated target gene expression. Expression patterns were compared between normal cervical tissue (SCSP0005605) and cervical cancer tissue (SCSP0005606). To systematically identify signaling pathways associated with these genes, gene set enrichment analysis (GSEA) was conducted based on the scRNA-seq expression profiles. Enriched pathways (FDR < 0.25) were prioritized based on the normalized enrichment score (NES).

### 2.9. Immune Microenvironment Correlation Analysis

Immune infiltration analysis was conducted using the TIMER 3.0 database (http://timer.cistrome.org/, accessed on 28 March 2026). Target genes (FASN and SCD1) were entered into the Immune Infiltration Analysis module with cervical cancer selected as the tumor type. Associations between gene expression and immune infiltration abundance were assessed by Spearman partial correlation; correlations with |r| > 0.3 and a two-tailed *p* value < 0.05 were deemed statistically significant. Using median expression as the cutoff, patients were divided into high and low subgroups, and the prognostic significance of immune infiltration was examined by survival analysis. Additionally, correlations between FASN/SCD1 and 60 immune checkpoint genes were analyzed using the Sangerbox 3.0 platform (http://sangerbox.com/, accessed on 28 March 2026). Spearman rank correlation coefficients were computed and visualized as a heatmap.

### 2.10. Statistical Analysis

Results are expressed as the mean ± SD of at least three independent experiments. Two-group comparisons were made with Student’s *t*-test; multi-group data were analyzed by one-way or two-way ANOVA followed by Tukey’s test. A *p* value < 0.05 was considered statistically significant. All calculations were carried out with GraphPad Prism 9.0.

### 2.11. Use of Generative AI Tools

Generative AI tools were used exclusively for language editing of this manuscript; no AI tools were involved in the design, execution, or analysis of the experiments. Specifically, DeepSeek (model version: DeepSeek-V4.1-Flash, Hangzhou DeepSeek Artificial Intelligence Basic Technology Research Co., Ltd., Hangzhou, China) was used for language polishing, grammar and spelling correction, and formatting assistance. All AI-assisted output was reviewed and edited by the authors, who take full responsibility for the content.

## 3. Results

### 3.1. Low-Dose DHC Synergistically Enhances the Cytotoxicity of PTX in Cervical Cancer Cells

**Figure 1:**
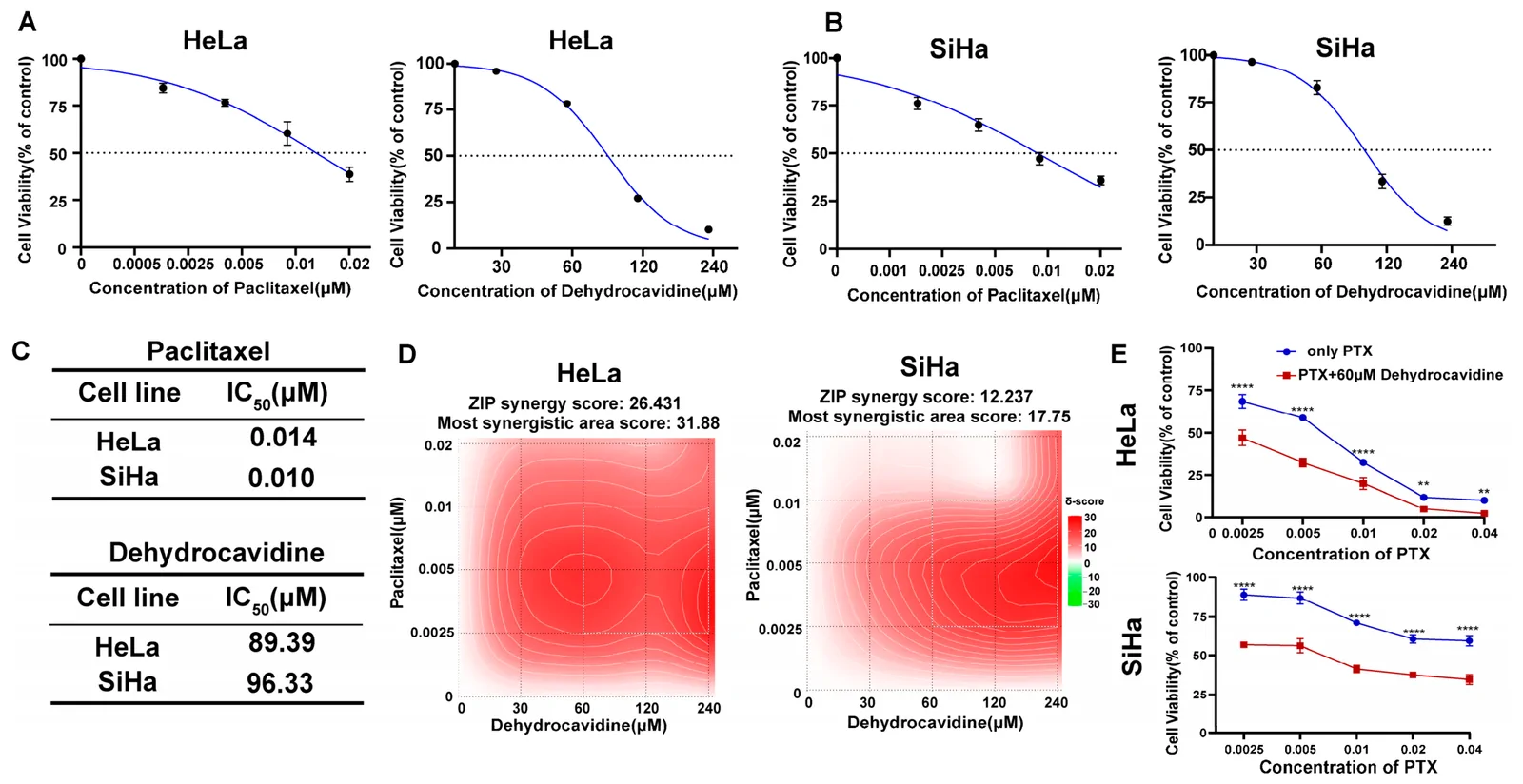
DHC synergistically enhances the cytotoxicity of low-dose PTX in cervical cancer cells. (**A**,**B**) Dose–response curves of PTX and DHC in HeLa (**A**) and SiHa (**B**) cells. Cells were exposed to the indicated concentrations of drugs for 48 h, and cell viability was determined by a CCK-8 assay. (**C**) IC_50_ of PTX and DHC in HeLa and SiHa cells. (**D**) ZIP synergy score heatmap of the PTX–DHC combination. (**E**) Viability of HeLa and SiHa cells treated with vehicle, PTX (0.0025 μM), DHC (60 μM), or their combination for 48 h. ** *p* < 0.01, **** *p* < 0.0001.

To evaluate the combined effect of PTX and DHC, we first examined the individual effects of each drug on the proliferation of HeLa and SiHa cells. Both drugs inhibited cell viability in a concentration-dependent manner (Figure 1A,B), and their IC_50_ values are summarized in Figure 1C. Based on the single-drug results, we further assessed the interactions of a series of PTX–DHC combinations using the ZIP synergy model. As shown in Figure 1D, the combination exhibited synergistic effects in both cell lines, with mean ZIP scores of 26.43 in HeLa cells and 12.24 in SiHa cells, both exceeding the threshold for strong synergy (δ > 10). From the synergy heatmap, we identified the region with the highest synergy scores. Within this region, we selected the combination containing the lowest PTX concentration (0.0025 μM), in line with our aim to minimize PTX-related toxicity while preserving synergy. Subsequently, we treated cells with this combination along with single-drug and vehicle controls. Treatment with this combination markedly reduced cell viability compared with single-drug treatments (Figure 1E), confirming that low-dose DHC synergistically potentiates the cytotoxicity of PTX in cervical cancer cells.

### 3.2. Low-Dose PTX Combined with DHC Inhibits the Proliferation and Migration of Cervical Cancer Cells

**Figure 2:**
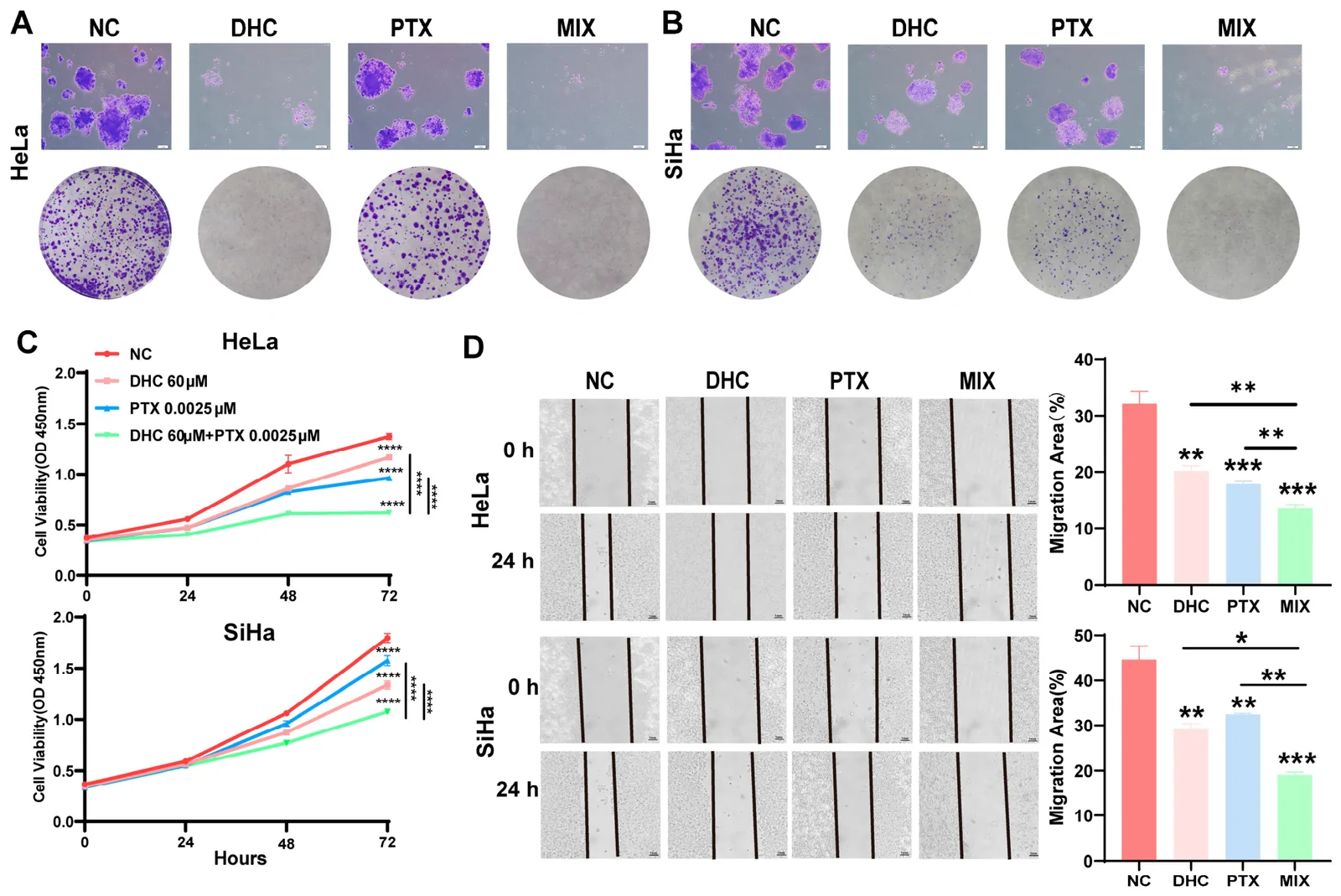
Low-dose paclitaxel combined with DHC inhibits the proliferation and migration of cervical cancer cells. (**A**,**B**) Colony formation assay showing the effects of different treatments on the colony-forming ability of HeLa (**A**) and SiHa (**B**) cells. Scale bars: 1 mm. (**C**) Relative viability of HeLa and SiHa cells after 24, 48, and 72 h of different treatments, assessed by CCK-8 assay. (**D**) Wound healing assay of HeLa and SiHa cells after 24 h of different treatments. Scale bars: 1 mm. * *p* < 0.05, ** *p* < 0.01, *** *p* < 0.001, **** *p* < 0.0001.

The long-term anti-proliferative effect of the combination was assessed by colony formation assay. Compared with the control group, PTX or DHC alone reduced colony number and density, whereas the combination nearly abrogated colony formation in both HeLa and SiHa cells (Figure 2A,B). Cell viability assays further showed that after 72 h, the combination reduced viability to 37.14% in HeLa cells and 55.54% in SiHa cells, significantly lower than either single-drug group (Figure 2C). In wound healing assays, the combination virtually abolished cell migration at 24 h, with migration rates significantly lower than those of single-drug groups (Figure 2D). Collectively, these data demonstrate that low-dose PTX plus DHC synergistically inhibits colony formation, viability, and migration of cervical cancer cells.

### 3.3. Network Pharmacology-Based Prediction of Potential Targets and Pathways of PTX Combined with DHC Against Cervical Cancer

**Figure 3:**
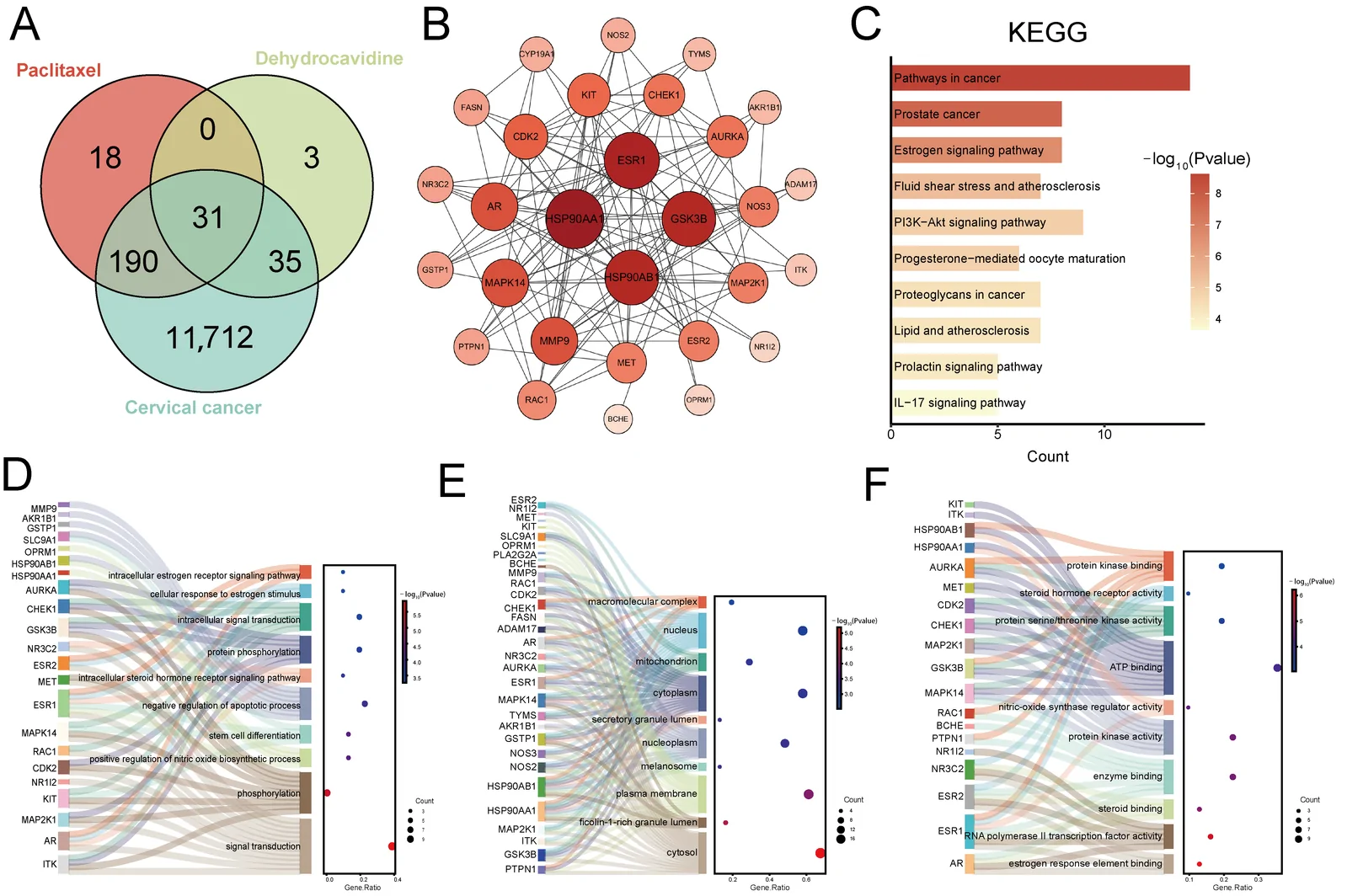
Network pharmacology analysis of PTX combined with DHC against cervical cancer. (**A**) Venn diagram showing the overlap among PTX targets, DHC targets, and cervical cancer-related genes, yielding 31 shared targets. (**B**) A STRING-based PPI network of the 31 shared targets is shown; nodes correspond to proteins and edges to their interactions. (**C**) Horizontal bar chart of KEGG pathway enrichment analysis. The horizontal axis shows the significantly enriched pathway terms, and the vertical axis represents the enrichment significance (−log_10_ *p* value), displaying the top-ranked pathways. (**D**–**F**) Sankey diagrams of GO enrichment analysis for BP, CC, and MF, respectively. Nodes on the left represent the intersecting targets, nodes on the right represent significantly enriched GO terms, and the connecting lines illustrate the mapping between targets and functions.

To preliminarily explore the molecular mechanisms underlying the anti-cervical cancer effects of low-dose PTX combined with DHC, we employed a network pharmacology approach for target prediction and pathway analysis. By integrating 11 databases, including TCMSP and HERB, we obtained 239 targets for PTX and 69 targets for DHC. Concurrently, 11,968 cervical cancer-related genes were collected from six databases, including TCGA and GeneCards. Venn diagram analysis revealed 31 intersecting targets shared among PTX, DHC, and cervical cancer (Figure 3A). The targets were subjected to STRING analysis for PPI network construction, followed by visualization with Cytoscape (Figure 3B). Pathway analysis using KEGG identified significant enrichment of the overlapping targets in several pathways, including lipid and atherosclerosis (Figure 3C). Based on GO annotation, the biological process (BP) category highlighted intracellular steroid hormone receptor signaling, cellular component (CC) analysis indicated predominant plasma membrane localization, and molecular function (MF) terms were dominated by steroid binding (Figure 3D–F), suggesting the involvement of these targets in steroid hormone-related biological processes.

**Figure 4:**
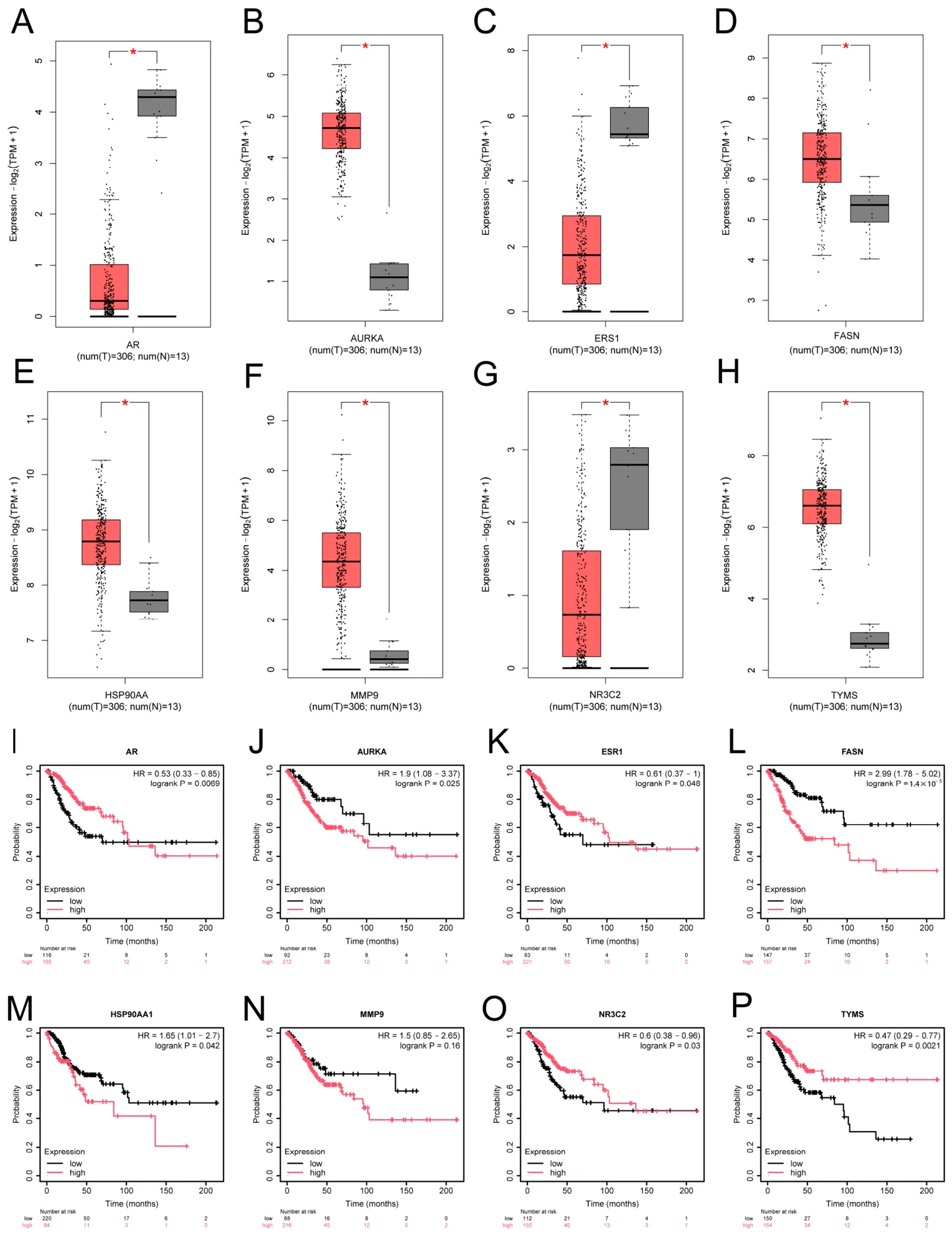
Differential expression and prognostic analysis of candidate targets of the combination treatment. (**A**–**H**) Differential expression of eight candidate genes (AR, AURKA, ESR1, FASN, HSP90AA1, MMP9, NR3C2, TYMS) between cervical cancer and normal tissues. (**I**–**P**) Kaplan–Meier overall survival curves of the eight genes in cervical cancer patients (Kaplan–Meier Plotter database). * *p* < 0.05.

Subsequently, differential expression analysis using TCGA data identified 16 significantly dysregulated genes among the 31 targets. Survival analysis via the Kaplan–Meier Plotter database showed that seven of these—ESR1, NR3C2, HSP90AA1, TYMS, FASN, AURKA, and AR—were significantly correlated with overall survival of cervical cancer patients. MMP9, although not significant, showed a trend toward poor prognosis and was included in subsequent validation. The expression patterns and survival curves of these eight genes are presented in Figure 4.

### 3.4. PTX Combined with DHC Exerts Anti-Cervical Cancer Effects by Suppressing Lipid Metabolism

**Figure 5:**
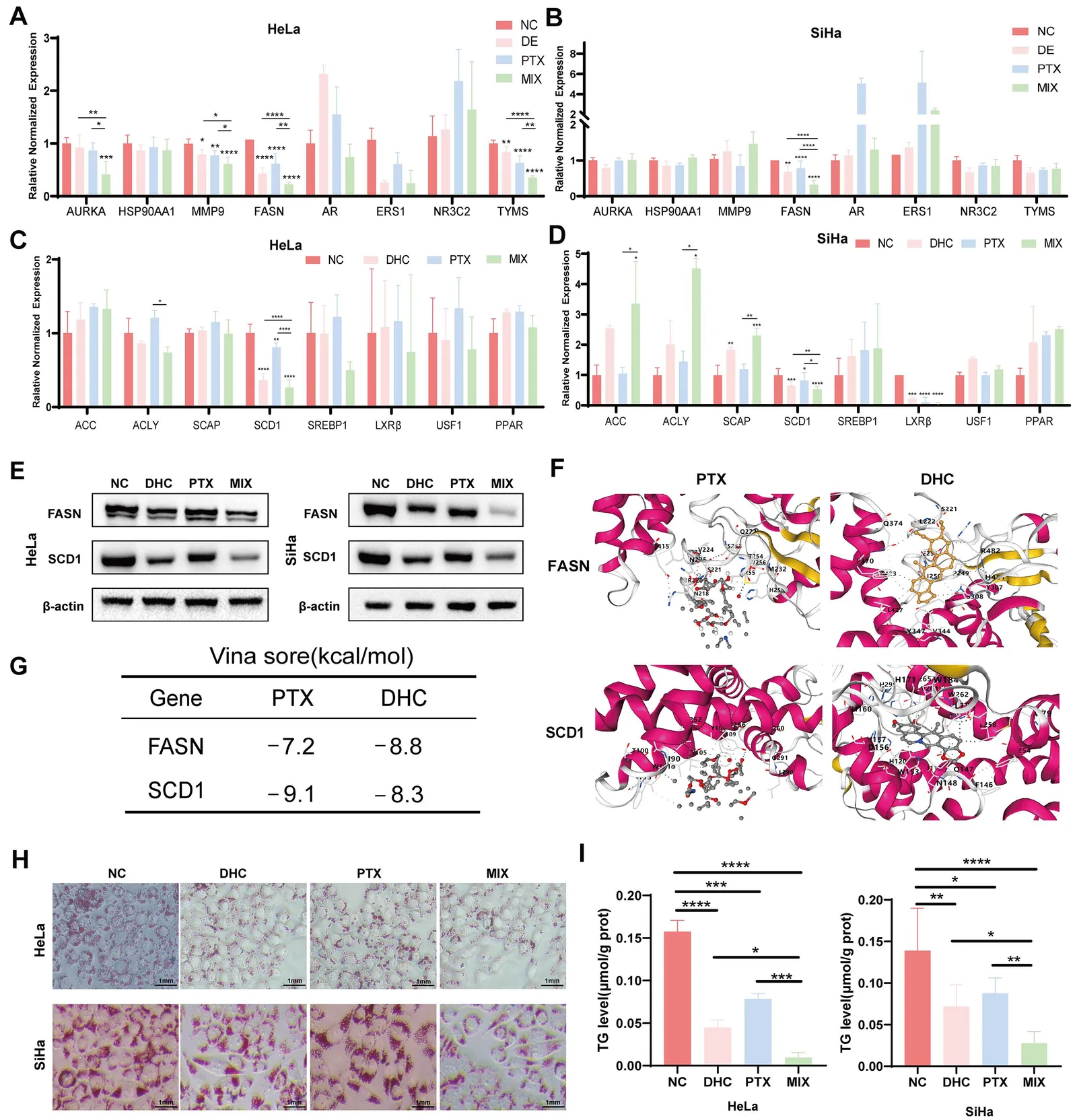
PTX combined with DHC suppresses cervical cancer cell growth through the lipid metabolism pathway. (**A**–**D**) RT-qPCR analysis of mRNA expression in HeLa (**A**,**C**) and SiHa (**B**,**D**) cells after 48 h of treatment. (**A**,**B**) Eight candidate target genes. (**C**,**D**) Eight lipid metabolism-related genes. (**E**) Western blot analysis of FASN and SCD1 protein expression in HeLa and SiHa cells, with β-actin as the loading control. (**F**) Molecular docking models of FASN and SCD1 with PTX and DHC. (**G**) Binding energies (kcal/mol) of PTX and DHC with FASN and SCD1 proteins. (**H**) Oil Red O staining showing intracellular neutral lipid droplets (scale bar, 1 mm). (**I**) Intracellular triglyceride (TG) content measurement. * *p* < 0.05, ** *p* < 0.01, *** *p* < 0.001, **** *p* < 0.0001.

Based on the network pharmacology predictions, RT-qPCR validation of the eight candidate targets revealed that only FASN was significantly downregulated by the combination in both HeLa and SiHa cells (Figure 5A,B), suggesting that FASN may serve as a key effector target of the combination therapy. Given that FASN is the rate-limiting enzyme of de novo fatty acid synthesis and is closely associated with lipid accumulation in tumors, we further expanded the screening to include eight lipid metabolism regulatory genes (ACC, ACLY, SCD1, SCAP, LXRβ, USF1, PPAR, and SREBP1) [18]. Among these, only stearoyl-CoA desaturase 1 (SCD1) showed significantly reduced mRNA levels following combination treatment (Figure 5C,D). Western blot analysis confirmed that the combination markedly reduced both FASN and SCD1 protein levels (Figure 5E).

Molecular docking indicated that PTX and DHC bind to FASN and SCD1 with binding energies below −5 kcal/mol, suggesting favorable binding affinities (Figure 5F,G). Subsequently, changes in lipid accumulation were evaluated by Oil Red O staining and triglyceride (TG) quantification. As shown in Figure 5H, the number of intracellular lipid droplets and the stained area were markedly reduced in the combination group, decreasing to 35.42% and 33.64% of the control in HeLa and SiHa cells, respectively, both significantly lower than those in the PTX alone group (40.89%) and the DHC alone group (62.68% in SiHa cells). TG measurement further corroborated these findings: in the combination group, TG content was 0.0096 and 0.0277 μmol/g protein in HeLa and SiHa cells, respectively, significantly lower than that in the control group (0.1576 and 0.1390 μmol/g protein) and the single-drug groups (PTX: 0.0784 and 0.0877 μmol/g protein; DHC: 0.0448 and 0.0719 μmol/g protein) (Figure 5I). Collectively, these results demonstrate that PTX combined with DHC inhibits lipid synthesis and accumulation in cervical cancer cells by downregulating FASN and SCD1 expression.

### 3.5. Single-Cell Sequencing-Based Expression Profiling of FASN and SCD1 and Lipid Metabolism Pathway Enrichment Analysis

**Figure 6:**
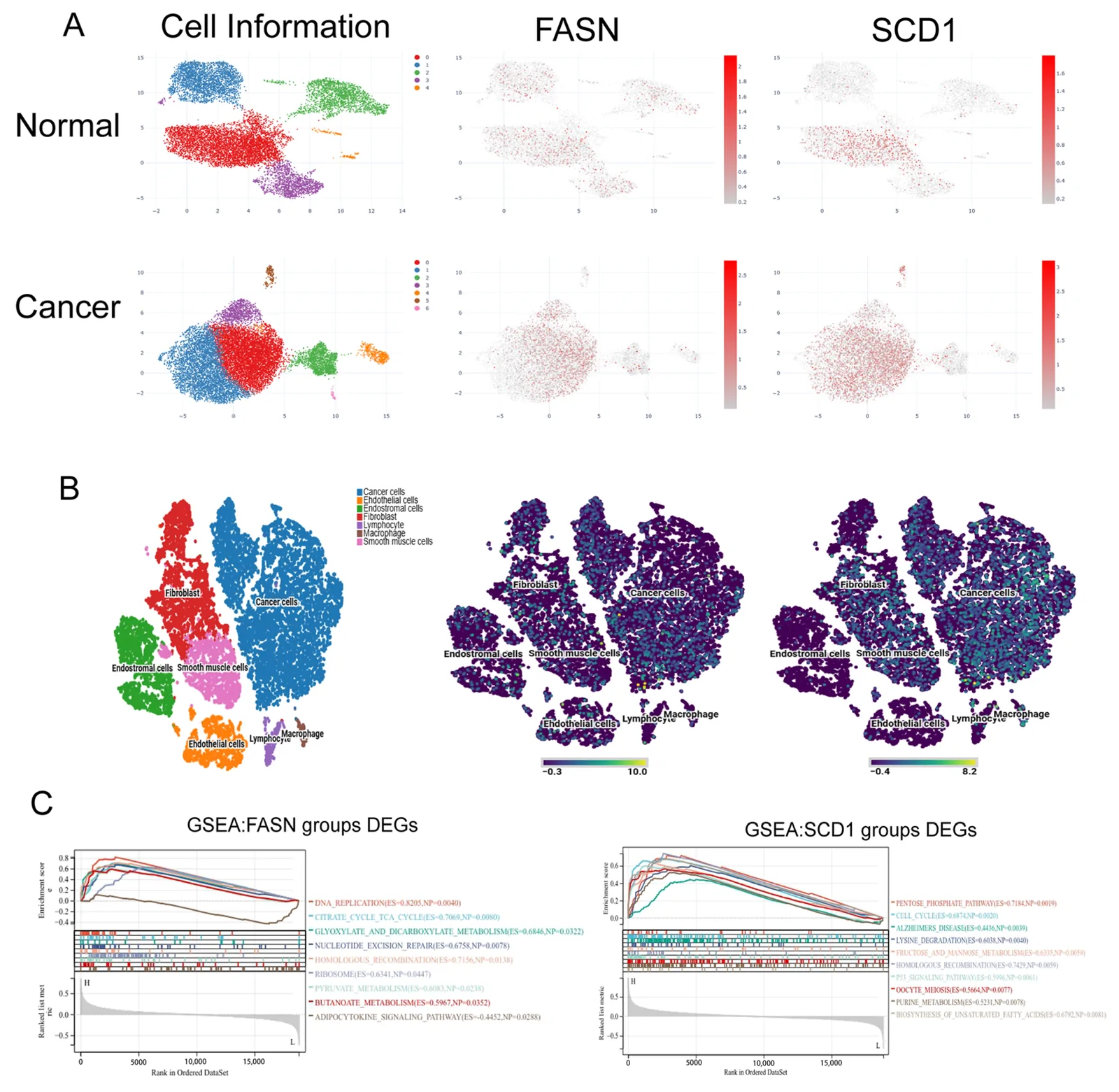
Single-cell expression characteristics and pathway enrichment analysis of FASN and SCD1 in cervical cancer. (**A**) Single-cell expression profiles of FASN and SCD1 in normal cervical tissue (SCSP0005605) and cervical cancer tissue (SCSP0005606). Color intensity indicates expression levels. (**B**) Expression distribution of FASN and SCD1 across different cell types in the cervical cancer tumor microenvironment (dataset VISDS000675). (**C**) GSEA enrichment analysis based on FASN and SCD1 expression levels, showing significantly enriched signaling pathways.

To characterize the expression patterns of FASN and SCD1 within the cervical cancer microenvironment, we first compared single-cell expression profiles between normal cervical tissue and cervical cancer tissue. The results revealed markedly elevated expression of both FASN and SCD1 in cervical cancer tissue compared with normal tissue (Figure 6A). Further analysis of cell-type distribution within the tumor microenvironment using dataset VISDS000675 showed that FASN was not only highly expressed in tumor cells but also broadly distributed across various stromal and immune cells, including fibroblasts, lymphocytes, and macrophages. In contrast, SCD1 expression was predominantly confined to tumor cells and fibroblasts (Figure 6B), suggesting that these two enzymes may play distinct roles in tumor–stroma interactions. To systematically decipher the downstream pathways regulated by FASN and SCD1, we performed GSEA. As shown in Figure 6C, FASN was enriched in the adipocytokine signaling pathway, while SCD1 was enriched in the biosynthesis of unsaturated fatty acids, further linking both genes to lipid metabolic remodeling.

### 3.6. Correlation Analysis of FASN and SCD1 Expression with the Immunosuppressive Microenvironment in Cervical Cancer

**Figure 7:**
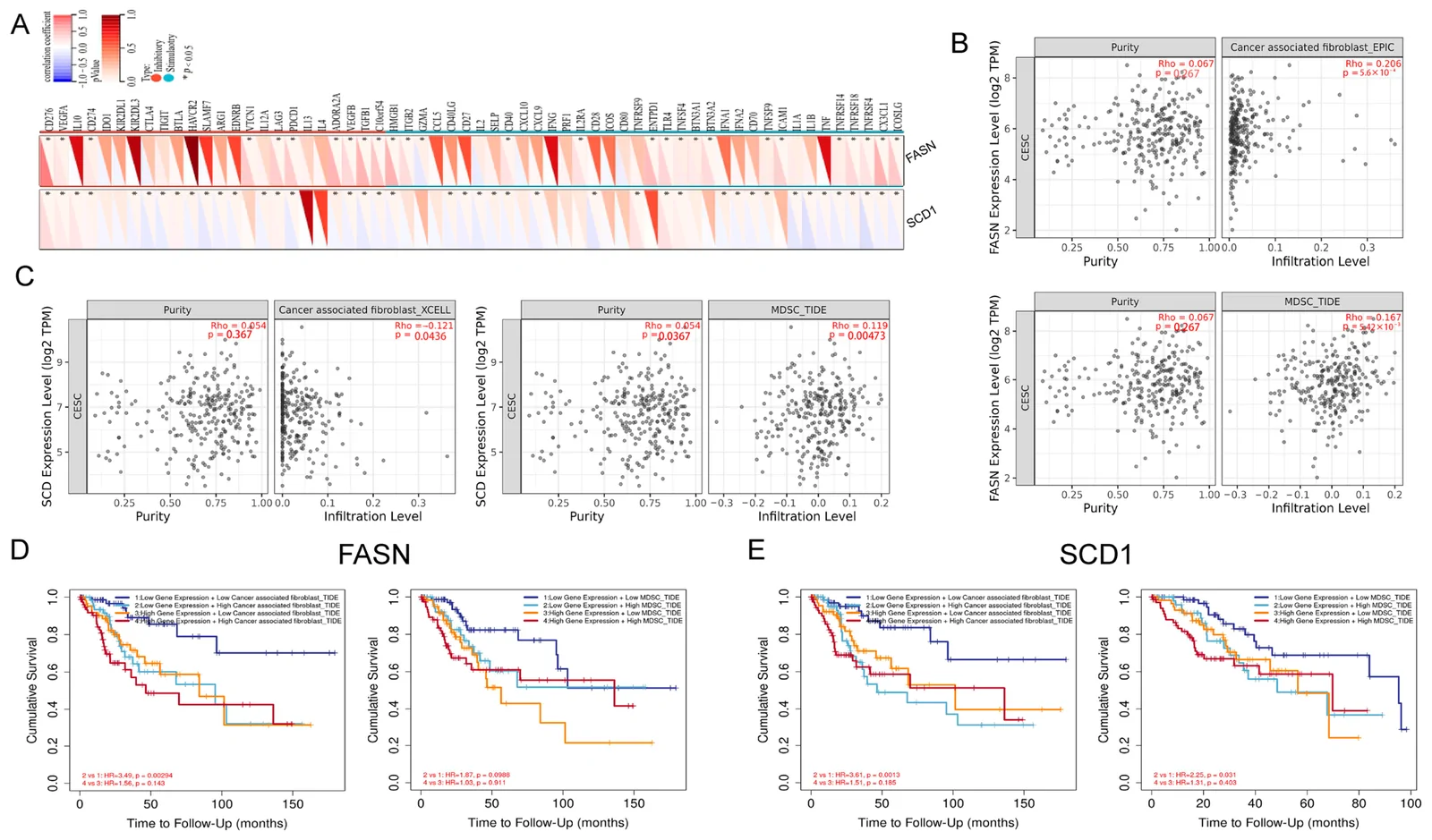
Correlation of FASN and SCD1 with the immunosuppressive microenvironment in cervical cancer. (**A**) Heatmap showing the correlations between FASN, SCD1, and immune checkpoint genes. Color blocks represent Spearman correlation coefficients (red, positive; blue, negative). (**B**,**C**) Analysis of the correlations between FASN (**B**) and SCD1 (**C**) expression and the infiltration levels of CAFs and MDSCs using the TIMER database. Y-axis: immune infiltration score. Gene expression is grouped by quartiles. Rho: partial correlation coefficient. (**D**) Kaplan–Meier overall survival curves stratified by FASN expression combined with CAF/MDSC infiltration. (**E**) Kaplan–Meier overall survival curves stratified by SCD1 expression combined with CAF/MDSC infiltration, analyzed as described in (**D**).

Our previous experiments demonstrated that PTX combined with DHC downregulates the expression of FASN and SCD1, and single-cell analysis further suggested their potential involvement in tumor microenvironment regulation. To elucidate their immunomodulatory functions, we first analyzed the correlations between FASN, SCD1, and 60 immune checkpoint genes using the Sangerbox 3.0 database. The heatmap revealed that FASN was significantly correlated with 15 immune checkpoints (14 positively, 1 negatively), while SCD1 was significantly correlated with 23 immune checkpoints (13 positively, 11 negatively) (Figure 7A). Further analysis of immune infiltration using the TIMER database showed that FASN expression was significantly positively correlated with the infiltration levels of both cancer-associated fibroblasts (CAFs) and myeloid-derived suppressor cells (MDSCs) (Figure 7B). In contrast, SCD1 expression exhibited a significant negative correlation with CAF infiltration (Rho = −0.121, *p* = 0.044) and a positive correlation with MDSC infiltration (Figure 7C). Combined survival stratification analysis demonstrated that, among patients with low FASN expression, high CAF infiltration (HR = 3.49, *p* = 0.003) or high MDSC infiltration (HR = 2.40, *p* = 0.033) significantly increased the risk of death. However, in the context of high FASN expression, the additional presence of high CAF or MDSC infiltration did not confer a significant incremental risk (*p* > 0.05) (Figure 7D), suggesting that high FASN expression may already sufficiently reflect the adverse prognostic effect of an immunosuppressive microenvironment. SCD1 exhibited a different pattern: patients with low SCD1 expression and high CAF infiltration had the poorest prognosis (HR = 3.61, *p* = 0.0013). Similarly, high MDSC infiltration significantly increased risk only in the low SCD1 expression group (HR = 2.25, *p* = 0.031), while its risk stratification effect disappeared in the high SCD1 expression group (Figure 7E). These results indicate that FASN and SCD1 participate in shaping the immunosuppressive microenvironment of cervical cancer through distinct pathways.

### 3.7. DHC Combined with Low-Dose PTX Enhances Growth Inhibition of HeLa Xenograft Tumors

**Figure 8:**
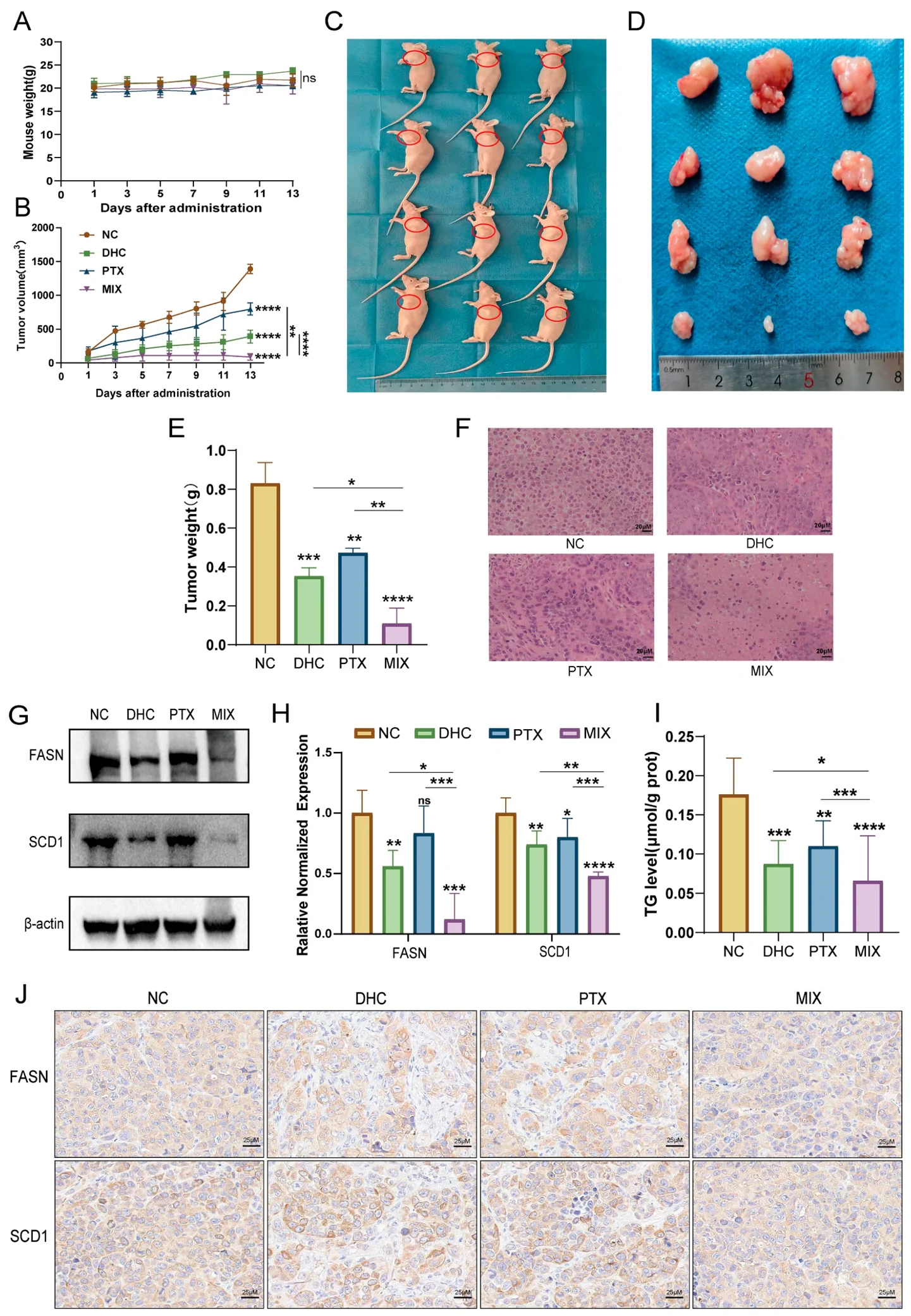
In vivo synergistic anti-tumor effects of PTX combined with DHC on HeLa xenograft tumors. (**A**) Body weight change curves. (**B**) Tumor volume growth curves. (**C**,**D**) Photographs of tumor-bearing mice (**C**) and excised tumors (**D**) at the treatment endpoint. (**E**) Final tumor weights. (**F**) H&E staining of tumor tissues (scale bar, 20 μm). (**G**) Western blot analysis of FASN and SCD1 protein levels, with β-actin as the loading control. (**H**) RT-qPCR analysis of FASN and SCD1 mRNA levels. (**I**) Triglyceride (TG) content in tumor tissues. (**J**) Immunohistochemistry detection of FASN and SCD1 protein expression and localization in tumor tissues (scale bar, 25 μm). ns, not significant. * *p* < 0.05, ** *p* < 0.01, *** *p* < 0.001, **** *p* < 0.0001.

To validate the in vitro synergistic effects in vivo, we established a subcutaneous xenograft model of HeLa cells in nude mice. Once tumors became palpable, the mice were randomly divided into four groups and intraperitoneally injected with saline (control), PTX (5 mg/kg), DHC (50 mg/kg), or the combination regimen every other day for 13 days. During the treatment period, the body weight of mice in all groups increased steadily without significant decline (Figure 8A), indicating that this regimen was well-tolerated at the tested dose. As shown in Figure 8B, tumor growth was markedly suppressed in the combination group compared with the control and single-drug groups. At the endpoint, the tumors were excised; the combination group exhibited markedly smaller tumors (Figure 8C,D), and the tumor weight was also significantly lower than that of the other three groups (Figure 8E).

Histological analysis revealed that tumor cells in the control group were densely arranged, whereas large areas of necrosis and markedly reduced cell density were observed in the combination group (Figure 8F). To determine the in vivo lipid metabolic changes, we examined the expression of FASN and SCD1 and the triglyceride content in tumor tissues. The combination significantly lowered FASN and SCD1 transcript levels compared with either PTX or DHC alone (Figure 8H). Western blot and immunohistochemistry analyses consistently confirmed that the combination led to a substantial reduction in FASN and SCD1 protein abundance (Figure 8G,J). Meanwhile, the triglyceride content in the tumor tissues of the combination group decreased to 0.02635 μmol/g protein, which was significantly lower than that in the control group (0.1364 μmol/g protein), the PTX alone group (0.04784 μmol/g protein), and the DHC alone group (0.07058 μmol/g protein) (Figure 8I). In summary, low-dose PTX combined with DHC effectively inhibited the growth of cervical cancer xenografts in vivo, and this effect was closely associated with the suppression of FASN/SCD1-mediated lipid synthesis.

## 4. Discussion

This study demonstrates that low-dose PTX (0.0025 μM) combined with DHC exerts potent synergistic anti-proliferative and anti-migratory effects in cervical cancer cells by coordinately downregulating the lipogenic enzymes FASN and SCD1, thereby suppressing lipid metabolism reprogramming. In vivo, this regimen significantly restrained xenograft tumor growth without inducing body weight loss, suggesting an acceptable tolerability profile at the tested dose. These findings offer a promising strategy to circumvent PTX resistance while potentially mitigating its dose-limiting toxicities, pending systematic toxicological evaluation.

As a first-line chemotherapeutic agent for cervical cancer, PTX is frequently compromised by neurotoxicity and acquired resistance [11,12]. A notable observation is that DHC at 60 μM—a concentration below its single-drug IC_50_ (approximately 89–96 μM)—markedly potentiated the cytotoxicity of PTX at 0.0025 μM (approximately one-fifth to one-sixth of its IC_50_). This sub-IC_50_ synergy implies that DHC sensitizes tumor cells to PTX, most likely through metabolic remodeling. Similar paradigms have been documented for other natural products: ginsenoside Rg5 reverses PTX resistance by inhibiting the PI3K/Akt/NF-κB axis [19], while kaempferol elevates intracellular PTX levels by blocking P-glycoprotein-mediated efflux [20]. Nevertheless, the molecular basis underlying DHC synergy in cervical cancer remains incompletely defined, and its cytotoxicity toward normal cells and therapeutic window warrant systematic investigation.

A central mechanistic insight of this work is that the combination substantially downregulates FASN and SCD1 and reduces intracellular lipid accumulation (Figure 5). FASN and SCD1 are key enzymes in de novo fatty acid synthesis and desaturation, respectively, and both are frequently overexpressed in cervical cancer and associated with poor prognosis [21,22,23,24,25,26]. Notably, pharmacological inhibition of FASN with the clinically safe inhibitor TVB-2640 has been shown to restore cisplatin sensitivity in cervical cancer by promoting SLC7A11-mediated ferroptosis, further supporting FASN as a promising therapeutic target in combination regimens [27]. Consistently, KEGG pathway enrichment analysis revealed that the 31 shared targets were significantly enriched in lipid and atherosclerosis, PI3K-Akt, and IL-17 signaling pathways (Figure 3C). The enrichment of the lipid and atherosclerosis pathway aligns well with our experimental evidence of FASN/SCD1 suppression and attenuated lipogenesis. Furthermore, FASN is transcriptionally governed by SREBP1, whose activity is under the control of the PI3K-Akt cascade [28]. These clues provide a framework for subsequent mechanistic dissection.

Previous investigations have largely focused on FASN and SCD1 as independent oncogenic drivers. Our study reveals an additional layer: a chemotherapy–natural product combination can simultaneously target two sequential lipogenic enzymes, depriving cancer cells of both energy supply and membrane building blocks. This dual metabolic blockade may explain the robust synergy: PTX arrests cells in mitosis by stabilizing microtubules [9], whereas DHC appears to sever metabolic compensatory mechanisms, generating a “synthetic lethal”-like effect [29,30]. Because FASN and SCD1 act sequentially, their co-suppression could synergistically amplify metabolic disruption. However, the precise mechanism by which the combination regulates FASN/SCD1 transcription or protein stability remains elusive. Future gene silencing or overexpression studies are required to determine whether these two enzymes are direct effectors. Single-cell analysis revealed that FASN and SCD1 were highly expressed in tumor cells (Figure 6) and significantly correlated with CAFs and MDSCs infiltration (Figure 7). This raises an intriguing question: does FASN/SCD1-mediated lipid metabolism reprogramming participate in shaping an immunosuppressive microenvironment? Tumor cell lipid remodeling can influence immune cell recruitment and function through lipid mediators or membrane lipid alterations [31,32]. Notably, the IL-17 signaling pathway, which was also enriched in our KEGG analysis, bridges lipid metabolism and immunosuppression: lipid mediators such as prostaglandin E2 promote Th17 differentiation and IL-17 secretion, and IL-17 in turn recruits MDSCs to facilitate immune escape [33]. Our immune infiltration analysis further linked FASN/SCD1 expression patterns to CAF/MDSC infiltration levels and patient prognosis (Figure 7). Therefore, the combination may not only directly suppress tumor cell proliferation by inhibiting FASN/SCD1 but also indirectly ameliorate the immune microenvironment by disrupting lipid-mediated IL-17 signaling and other immunoregulatory pathways. Given the emerging role of FASN in immune cell metabolism and evasion, future studies should examine this combination on specific immune cell subsets, such as T cells, macrophages, and MDSCs [34], using co-culture systems or humanized immune-competent models.

Several limitations should be acknowledged. First, our data demonstrate a consistent correlation but not a direct causal link between FASN/SCD1 downregulation and anti-tumor effects; overexpression or knockdown experiments are needed. Second, the employment of immunodeficient nude mice precludes evaluation of adaptive immune responses; future studies in immune-competent models are imperative to validate the immunomodulatory dimension. Third, tolerability was only preliminarily assessed by body weight monitoring; comprehensive pharmacokinetic, toxicological, and normal cell cytotoxicity profiling is a prerequisite for clinical translation. Fourth, we acknowledge that the in vivo experiment included only three mice per group, which limits the statistical power and generalizability of the findings. This sample size was chosen for an initial efficacy assessment and to minimize animal use in accordance with the 3R principles; however, larger cohorts are necessary to confirm the reproducibility of the observed anti-tumor effects. Finally, the pharmacokinetic properties, tissue distribution, and metabolism of DHC itself remain largely unexplored and require dedicated investigation.

## 5. Conclusions

**Figure 9:**
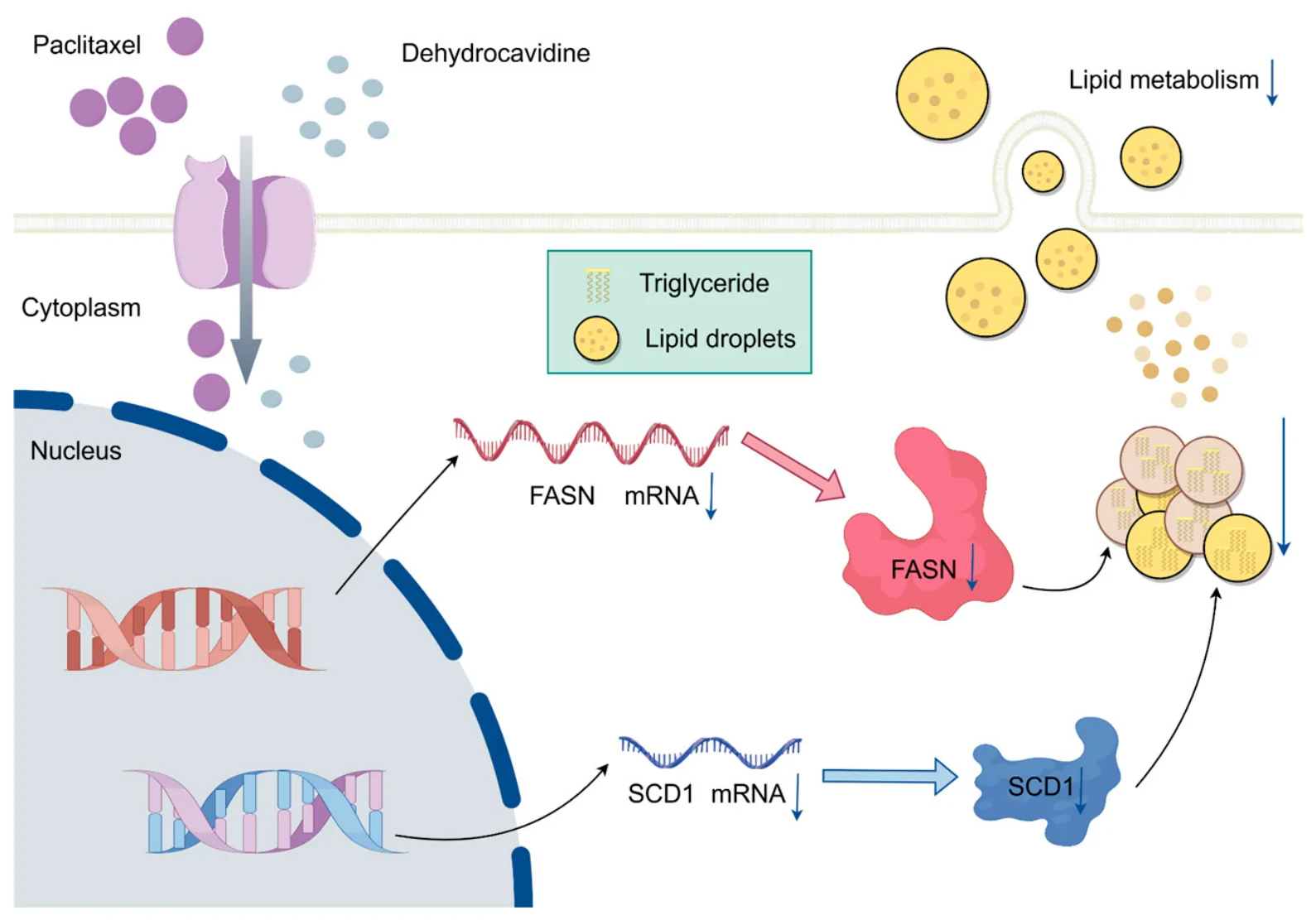
Schematic illustration of the synergistic anti-cervical cancer mechanism of DHC combined with PTX. Low-dose PTX combined with DHC downregulates the expression of FASN and SCD1, inhibits fatty acid synthesis and desaturation, reduces lipid droplet and triglyceride accumulation, and thereby synergistically suppresses the proliferation, migration, and in vivo tumor growth of cervical cancer cells.

In summary, this study demonstrates that low-dose PTX combined with DHC synergistically inhibits proliferation, migration, and in vivo tumor growth of cervical cancer cells by downregulating FASN and SCD1 and suppressing lipid droplet and triglyceride accumulation (Figure 9). This combination strategy provides a novel approach to overcome PTX resistance and reduce chemotherapy-associated toxicity, and lays a foundation for targeting lipid metabolism in cervical cancer. Future work should focus on elucidating the upstream regulatory mechanisms and validating the translational potential of this regimen in more clinically relevant models.

## Data Availability

The experimental data produced in this work are contained within the manuscript. Publicly accessible datasets utilized in this study were sourced from the Cell-omics Data Coordinate Platform (CDCP), GEPIA (integrating TCGA and GTEx), Kaplan–Meier Plotter, TIMER 3.0, and Sangerbox 3.0, as described in Section 2.

## Supplementary Materials

The following supporting information can be downloaded at https://www.mdpi.com/article/10.3390/biom16091354/s1, Table S1: Primer sequences for RT-qPCR. Figures S1–S3 are original images of Western Blot in Figure 5 and Figure 8.
