# Supplementary material for: Low-Dose Paclitaxel Combined with Dehydrocavidine Synergistically Suppresses Cervical Cancer by Inhibiting FASN/SCD1-Mediated Lipid Metabolism
Source: Biomolecules. 2026 Sep 17;16(9):1354. doi: 10.3390/biom16091354 (PMC13604622; doi:10.3390/biom16091354)
Supplement: Supplementary file 1 [file biomolecules-16-01354-s001.zip › biomolecules-4528202-supplementary table.pdf]

Table S1. Primer sequences for RT-qPCR.

Gene	Forward primer (5′–3′)	Reverse primer (5′–3′)
AR	CTCTCACATGTGGAAGCTGC	AGCCTCTCCTTCCTCCTGTA
AURKA	GTGTTCTCGTGACTCAGC	CTGATTCTGAACCGGCTTG
ESR1	CCAACCAGTGCACCATTGAT	TTTGATCATGAGCGGGCTTG
HSP90AA1	CATAACGATGATGAGCAGTACGC	GACCCATAGGTTCACCTGTGT
MMP9	GCGTCTTCCCCTTCACTTTC	ATAGGGTACATGAGCGCCTC
FASN	GTTCATCTGCTCTGGGATGG	TGTCATCAAAGGTGCTCTCG
NR3C2	CCTGCTTGCAGACTTCAG	GCTCGTGAGATTGTGGAG
TYMS	ATCATGTGCGCTTGGAATCC	CGCAATCATGTACGTGAGCA
ACC	CAATCTTGAGGGCTAGGTCT	TGAATCGAGAGTGCTGGTTC
ACLY	GGACTTCGGCAGAGGTAGAG	TGAACCGATTCTGGATGGCT
SCAP	GACTTCGGCTGAGGATGAC	GGTAGCAGCAGGCTAAGAT
SCD1	GCAGGACGATATCTCTAGCT	GGCATCGTCTCCAACTTATC
SREBP1	TCTGGAGGCATCGCAAGC	GAGGTTCCAGAGGAGGCTAC
LXRβ	CACGAGACAGAGTGTATCAC	GAGAAGATGTTGATGGCGAT
USF1	GGACAGTGCAGATTCAGGAAG	CATTCTCAGTTCGGAAGACGT
PPAR	GTGGATCTCTCCGTAATGG	GGAATGTCTTCGTAATGTGG
GAPDH	CACATCGCTCAGACACCATG	TGACGGTGCCATGGAATTTG
