# Supplementary material for: Low-Dose Paclitaxel Combined with Dehydrocavidine Synergistically Suppresses Cervical Cancer by Inhibiting FASN/SCD1-Mediated Lipid Metabolism
Source: Biomolecules. 2026 Sep 17;16(9):1354. doi: 10.3390/biom16091354 (PMC13604622; doi:10.3390/biom16091354)
Supplement: Supplementary file 1 [file biomolecules-16-01354-s001.zip › biomolecules-4528202-WB.pdf]

Figure S1 HeLa

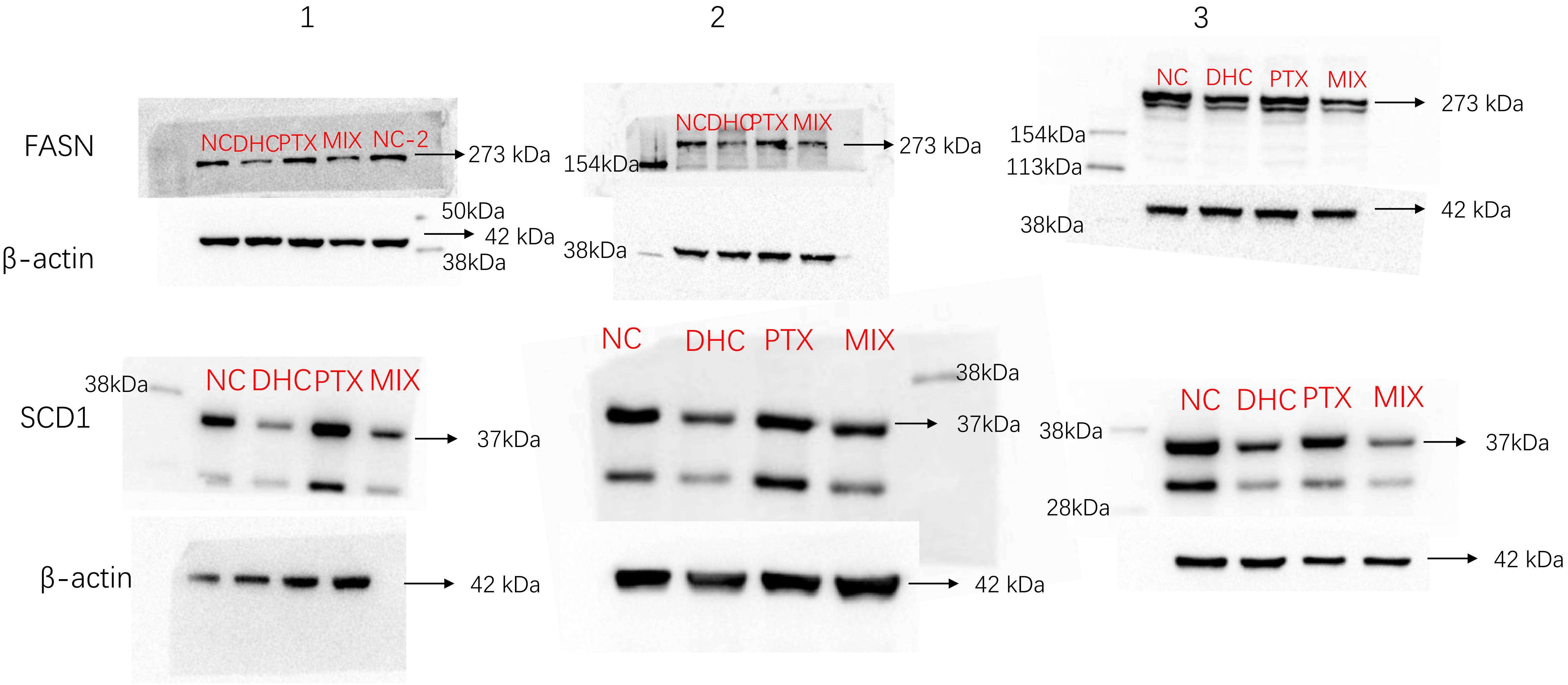

Figure S2 SiHa

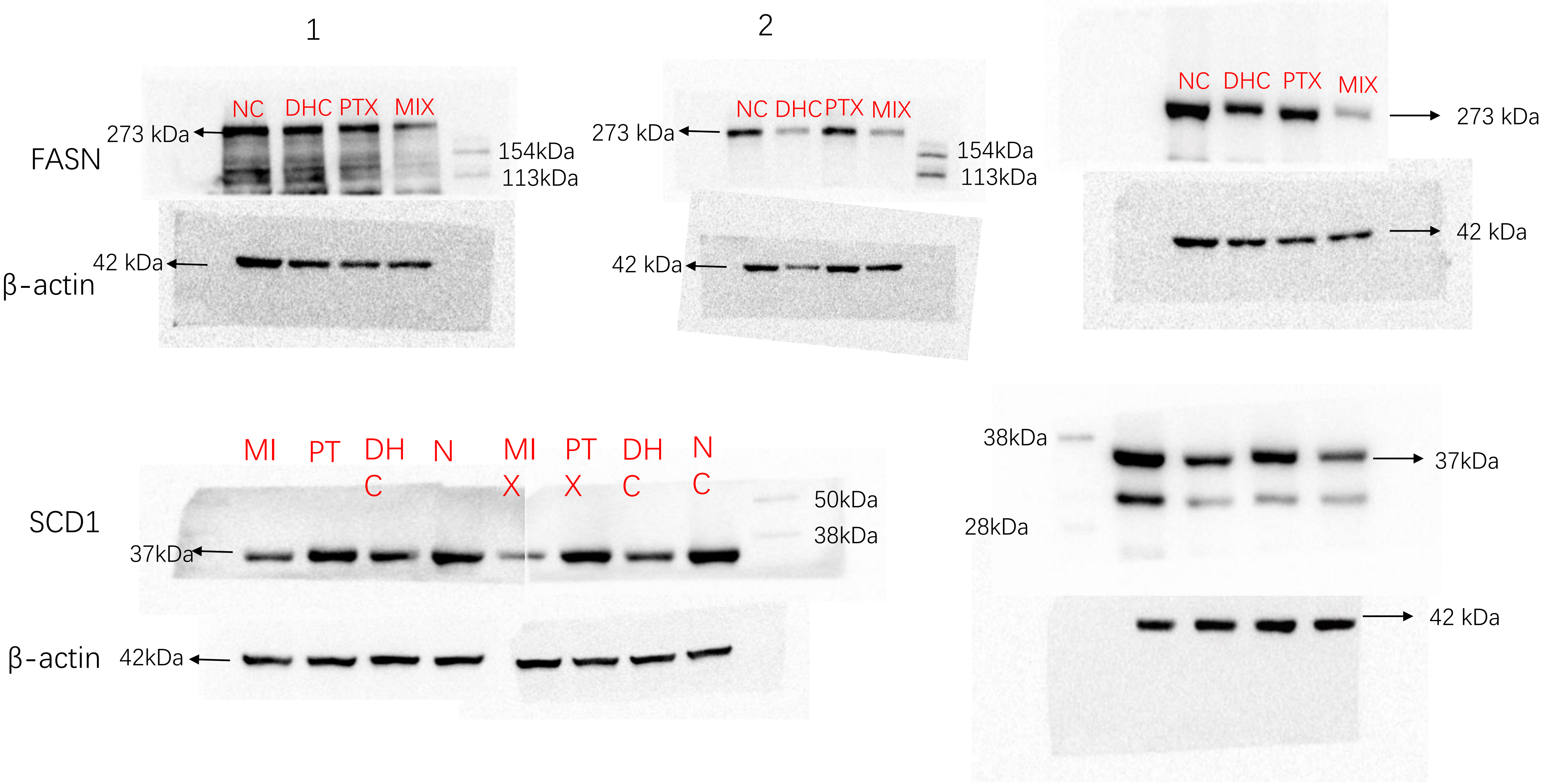

Figure S3

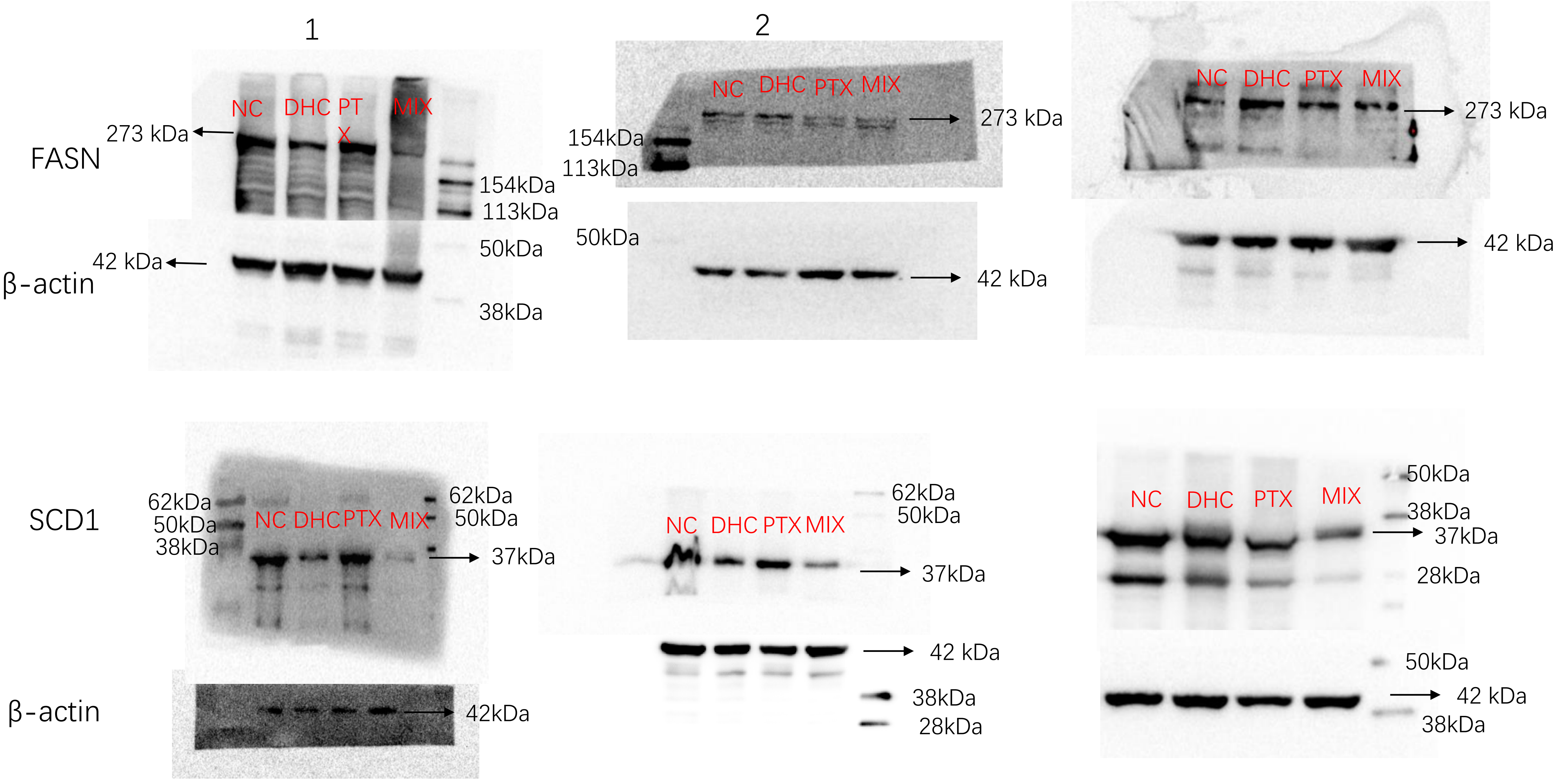
1
2
3
FASN
273 kDa
NC
DHC
PT
X
MIX
154kDa
113kDa
50kDa
38kDa
42 kDa
β-actin
NC DHC PTX MIX
273 kDa
154kDa
113kDa
50kDa
42 kDa
NC DHC PTX MIX
273 kDa
42 kDa
SCD1
62kDa
50kDa
38kDa
NC DHC PTX MIX
62kDa
50kDa
37kDa
β-actin
42kDa
NC DHC PTX MIX
62kDa
50kDa
37kDa
42 kDa
38kDa
28kDa
NC DHC PTX MIX
50kDa
38kDa
37kDa
28kDa
50kDa
42 kDa
38kDa
